# The longitudinal effects of maternal parenting practices on children’s body mass index z-scores are lagged and differential

**DOI:** 10.1186/s12887-023-03902-9

**Published:** 2023-05-29

**Authors:** Lisa Kakinami, Prince Kevin Danieles, Fatemeh Hosseininasabnajar, Tracie A. Barnett, Mélanie Henderson, Andraea Van Hulst, Lisa A. Serbin, Dale M. Stack, Gilles Paradis

**Affiliations:** 1 Department of Mathematics and Statistics, Concordia University, 1455 de Maisonneuve Blvd West, Montréal, QC H3G 1M8 Canada; 2 PERFORM Centre, Concordia University, Montréal, QC Canada; 3 Department of Family Medicine, McGill University, Montréal, QC Canada; 4 Centre de Recherche du CHU Sainte-Justine, Montréal, QC Canada; 5 Department of Pediatrics, Université de Montréal, Montréal, QC Canada; 6 School of Public Health, Department of Social and Preventive Medicine, Université de Montréal, Montréal, QC Canada; 7 Ingram School of Nursing, McGill University, Montréal, QC Canada; 8 Department of Psychology, Concordia University, Montréal, QC Canada; 9 Centre for Research in Human Development, Montréal, Canada; 10 Department of Epidemiology, Biostatistics, and Occupational Health, McGill University, Montréal, QC Canada

**Keywords:** Obesity, Longitudinal, Bidirectional, Parenting practices, Body mass index z-scores

## Abstract

**Background:**

The longitudinal relation between parenting practices and styles with children’s body mass index z-scores (zBMI) is poorly understood. Previous studies suggest the relationship may be complex, but small samples and short follow-ups diminish the strength of the evidence. The objectives of this study were to investigate whether the relationship is bidirectional, time-varying, and lagged using data from a large, representative birth cohort of Quebec children.

**Methods:**

Data were from the Québec Longitudinal Study of Child Development (QLSCD)*,* a prospective birth cohort (*n* = 1,602). The mothers’ interactions with their children (at ages 6, 8, 10, and 12 years) were utilized in factor analysis to identify three latent parenting practices (disciplinarian, lenient, and responsive). The parenting practices were analyzed with K-means clustering to identify the parenting styles. The temporal and bidirectional relationships were assessed in a cross-lagged path analysis using a structural equation modelling framework. Mixed models controlling for age, sex, income, mother’s education, and whether the participant was first-born were estimated. Missing data were handled with full information maximum likelihood.

**Results:**

From the linear mixed models, greater lenient and responsive parenting practices were associated with higher zBMI (B = 0.03, *p* < 0.05) two years later. However, there was no evidence that the relationship was bidirectional nor that parenting style was predictive of children’s zBMI.

**Conclusion:**

While mothers’ parenting practices were unaffected by their children’s zBMI, parental practices were predictive of future zBMI among their prepubertal children. More in-depth exploration of parenting practices and their potential impact on pediatric weight is needed.

## Introduction

Approximately 30% of Canadian children are living with overweight or obesity [1]. These children are more likely to suffer from dyslipidemia, hyperinsulinaemia, high blood pressure, and other chronic medical conditions during childhood [2, 3]. They are also significantly more likely to become adults with obesity [4]. Because nearly ¼ of Canadian children already have overweight or obesity when they enter preschool [5], a better understanding of the early childhood environment is needed to understand the social and familial contexts that are associated with excess adiposity. In particular, parenting practices and parenting styles may provide insights that could inform prevention efforts. Parenting practices are specific behaviours and interactions between parent and child and occur along a continuum (such as the degree to which parents punish their child for misbehaving). In contrast, parenting styles group the constellation of parenting practices into mutually-exclusive classifications [6].

Of the four largely agreed upon general parenting styles (authoritative, authoritarian, permissive, and uninvolved), the authoritative parenting style is consistently associated with successful socialization and cognitive development in children [7, 8]. However, there are considerable gaps in our understanding of how parenting styles affect physical health outcomes. While cross-sectional studies consistently report an association between authoritative parenting style and lowest body mass index (BMI) z-scores, the longitudinal relationships are less clear [9–14]. Additionally, preliminary evidence suggests that there is a bidirectional, dynamic interrelationship between parenting and child weight: children with obesity are described as having a more difficult temperament than non-obese children by their parents [15, 16], which in turn may affect the parents’ parenting styles. While the underlying parenting practices occur along a continuum varying in demandingness (the degree to which parents set expectations and enforce boundaries) and responsiveness (the degree to which parents demonstrate warmth and affection), parenting styles classify these parenting practices into four mutually exclusive parenting styles. These categorizations overlook the variation of parenting practices within a parenting style, which may be further compounded when exploring the relationships over time. Thus, investigating the underlying parenting practices may reveal important longitudinal information that the parenting style categorizations may overlook. The primary objective of this study was therefore to investigate the bidirectional, longitudinal relationship between parenting practices and children’s body mass index (BMI) z-scores. A secondary objective was to investigate the bidirectional, longitudinal relationship between parenting styles and children’s BMI z-scores.

## Materials and methods

This study utilized data from the Quebec Longitudinal Study of Child Development (QLSCD), a large, prospective birth cohort initiated in 1998 (*n* = 2,120) with 13 waves of data collection over 16 years. The study methodology is previously published [17] and briefly described here. The QLSCD cohort was initiated by the Institut de la statistique du Québec, and is a representative sample of singleton births in Quebec. Children were identified from the 1997–1998 Quebec live birth registry. Those born before 24 or after 42 weeks’ gestation, of unknown gestational age, or from Cree and Inuit territories were excluded (~ 4% of live births). The remaining target population was randomly sampled using a multistage cluster sampling design resulting in a sample of 2,120 children. Data were collected annually (1998–2005) and biennially (2005-) in the participant’s home (16 years of follow-up, in 13 data collection waves). The person who was most knowledgeable about the child (> 95% the mother) completed self-administered questionnaires and interviews with trained study staff. For this secondary data analysis, only the data collection waves in which either the main predictors of interest (parenting practices or parenting styles as described further), or the outcome of interest (BMI z-scores) were collected were used. The study was approved by the Direction Santé Quebec of the Institut de la statistique du Quebec and the Université de Montréal ethics committees. Parents signed informed consent and children provided verbal assent. This secondary data analysis was approved by Concordia University’s (#30013495) ethics committee.

### Parenting practices

When the child was aged 6, 8, 10, and 12 years, mothers were asked 14 questions which assessed whether their interactions with their child were positive (e.g., “How often did you do something special with him/her that he/she enjoys”), negative (e.g., “How often did you get angry when you were punishing him/her”), or lenient (e.g., “How often did he/she get away with things you felt should have been punished”) on five-point Likert scales (response options: never, less than half the time, about half the time, more than half the time, all the time). These were adapted sub-items from a reliable, internally consistent pre-existing measure of parenting practices [18].

In order to identify the parenting practices, the mother–child relationship items were entered into an exploratory factor analysis separately for each data collection wave. Due to the correlated nature of the factors, a promax rotation was used. Items with factor loadings < 0.40 were excluded, resulting in a final set of 10 items. From the factor analysis, three latent parenting practice factors based on eigenvalues above one were identified: (a) physical disciplinarian behaviours (e.g.: “How often did you grab firmly or shake your child when he/she was difficult?, *n* = 3 items), (b) leniency (e.g.: “When your child broke the rules or did things that he/she was not supposed to, how often did you: ignore it, do nothing?”, *n* = 4 items), and (c) responsiveness (e.g.: “How often do you and your child talk or play with each other, focusing attention on each other for five minutes?”, *n*= 3 items) [19].

### Parenting style

Starting with the factor scores, a scaled score was calculated for each parenting practice, and a cluster analysis grouped similar observations together. Different clustering methods were tested such as hierarchical methods (e.g., complete and Ward) and k-means. The agglomerative coefficient was used to compare these different hierarchical methods. While the Ward method was identified initially as the best hierarchical clustering method, it was inferior to k-means based on their average silhouette widths. Thus we used k-means clustering for each data collection wave. The labeling of the clusters *(authoritative, authoritarian, permissive, uninvolved) *were based on their methodological descriptions in the literature [7, 19]. For instance, the ‘permissive’ parenting style was identified as the cluster with the lowest average disciplinarian parenting practice, and highest permissive parenting practice.

### Body mass index (BMI)

Children’s height and weight were directly measured by trained staff when the children were approximately 6, 8, 10, and 12 years of age. Height was measured to the nearest 0.1 cm with a standard measuring tape, and weight to the nearest 0.1 kg with a calibrated spring scale. Both were measured in duplicate, and if the first two measurements differed by more than 0.5 cm (for height) or 0.2 kg (for weight), a third measurement was collected. The average of the two closest measurements was used to calculate and compare BMI to same-age and same-sex growth curves [20]. The child’s BMI was converted to z-scores using the World Health Organization growth curves [20]. Whether the child had obesity (BMIz-score of > 2) or whether the child had overweight or obesity (BMI z-score of > 1) as outcomes were assessed in a sensitivity analysis.

### Covariates

Covariates included: the child’s sex, age, whether the child was the first-born in the family, whether the mother had at least a high school diploma at baseline, and whether the household was currently considered low-income. Income was based on the mothers’ reports of total family income for the previous year and was compared to low-income cut-offs as defined by Statistics Canada (which adjusts for geographic region and size of the household) [21]. All variables (with the exception of mother’s education at baseline, whether the child was the first born, and the child’s sex) were treated as time-varying.

### Statistical analysis

Analyses were conducted with SAS 9.4 and MPLUS 8.1. Cronbach’s alphas were assessed for each factor score at each wave, with reliability assessed in accordance with the literature [22]. Whether parenting practices were time-varying was assessed with the intra-class correlation coefficient (ICC) by Shrout-Fleiss [23]. Based on the factor scores, the temporal and bidirectional relationships between the parenting practices, and children’s BMI z-scores were assessed using cross-lagged path analyses in a structural equations modelling framework. All analyses adjusted for the covariates previously described. Missing data were handled with full information maximum likelihood (FIML). As FIML preserves all observations (even when data are missing), analyses were restricted to participants who had at least one BMI z-score and at least one mother–child relationship measure completed within the waves regarded (*n* = 1,602).

Although the bidirectionality of parenting styles and BMIz over time was explored in path analysis, the covariance matrix was not positive definite and interpretation of results would be inappropriate. We therefore additionally assessed the bidirectionality between parenting styles and the risk of having overweight or obesity. However, parenting styles were not significant for predicting risk of overweight/obesity in the bidirectional model (data not shown), and we encountered the covariance matrix warning in the lagged longitudinal model. Thus we present results only for a continuous outcome (BMIz) as our dependent measure of interest.

Lastly, to explore both concurrent and intertemporal associations of parenting practices (measured at ages 6, 8, 10, 12 yrs) and the outcome (BMIz: ages 6, 8, 10, 12 yrs), two separate mixed models with FIML were tested. The first model maintained parenting practices as time-varying and examined the relationship within the same wave. The second model tested for parenting practices’ lagged effects on BMIz two years later. These models were repeated with parenting styles as the primary predictor of interest. Consistent with conventional lagged models where the number of time points originally available is reduced by the number of lags employed, the first and last data collection waves were omitted. Results were pooled for the final parameter estimates.

## Results

Characteristics of the study sample are presented (Table 1). Approximately 51% were female, and mean BMI z-scores at age 6, 8, 10, and 12 yrs were 0.17, 0.19, 0.58, and 0.57, respectively. All factor scores for parenting practices (physical disciplinarian behaviours, *n* = 3 items; leniency, *n* = 4 items, and responsiveness, *n* = 3 items had acceptable psychometric properties (α > 0.60) except the permissive parenting practice at age 10 and 12 waves (Cronbach’s α of 0.58 and 0.50, respectively). As the parenting practices were identified from factor scores, only standardized values are produced (mean values of 0) at each wave. However, the ICC for parenting practices was moderate (range: 0.37–0.49), suggesting that the underlying parenting practices were time-varying. The largest proportion of parenting styles at each wave was consistently authoritative (approximately 40%). Because the parenting styles were identified based on their relative cluster scores of the underlying parenting practices, the alignment between parenting practices and parenting styles were consistent with each other over time by definition. For instance, parents who had an authoritarian parenting style were by definition, those with low responsiveness, low leniency, and high disciplinary behaviours. However, the mean scores of parenting practices in parenting styles changed over time, reflecting the time-dependent nature of these behaviours based on the age of the child (data not shown).

**Table 1:** Descriptive statistics (*n* = 1,602)^a^

	Age 6	Age 8	Age 10	Age 12
Females^b^	51%			
First-born^b^	41%			
No post-secondary education (mother)^b^	43%			
Age	6.15 (0.25)	8.15 (0.26)	10.15 (0.26)	12.14 (0.25)
zBMI	0.17 (1.15)	0.19 (1.25)	0.58 (1.17)	0.57 (0.20)
With obesity (BMIz-scores >2)	20%	23%	33%	34%
Below the low-income thresholds	16%	15%	11%	13%
Parenting style				
Authoritarian	22%	20%	11%	8%
Authoritative	39%	42%	41%	41%
Uninvolved	20%	23%	29%	36%
Permissive	19%	15%	19%	15%

^a^Mean (SD) unless indicated otherwise

^b^Measured at baseline

Estimates for testing the cross-lagged bidirectional structural equation model are provided (Table 2); fit statistics were good (RMSEA = 0.052, CFI = 0.929, SRMR = 0.032). Leniency when the child was 6 years of age was marginally associated with higher zBMI two years later (B = 0.07, *p* = 0.05), as was the responsiveness when the child was 8 years of age (B = 0.06, *p* = 0.01). In contrast, using strong disciplinarian behaviours when the child was 8 years of age was associated with lower zBMI two years later (B = -0.06, *p* = 0.01). However, when assessing the bidirectional relationship between children’s BMI z-score on parenting practices, only zBMI at age 6 yrs was marginally associated with responsiveness two years later (B = 0.03, *p* = 0.06).

**Table 2:** Bidirectional cross-lagged (2-years) relationships between parenting practices and children’s BMI z-scores from a structural equation model

**Lagged effect of parenting practices on zBMI**			
	zBMI at age 8 B (SE)	zBMI at age 10 B (SE)	zBMI at age 12 B (SE)
**Disciplinarian**	-0.005 (0.030) *p* = 0.86	-0.055 (0.022) *p* = 0.01	-0.015 (0.018) *p* = 0.39
**Lenient**	0.066 (0.033) *p* = 0.05	0.039 (0.022) *p* = 0.09	-0.020 (0.017) *p* = 0.25
**Responsive**	0.024 (0.031) *p* = 0.44	0.056 (0.022) *p* = 0.01	0.001 (0.017) *p* = 0.96
**Lagged effect of zBMI on parenting practices**			
	Disciplinarian two years later B (SE)	Lenient two years later B (SE)	Responsive two years later B (SE)
**zBMI at age 6**	0.020 (0.020) *p* = 0.32	-0.008 (0.020) *p* = 0.68	0.034 (0.019) *p* = 0.06
**zBMI at age 8**	0.025 (0.017) *p* = 0.13	-0.002 (0.019) *p* = 0.92	-0.016 (0.016) *p* = 0.34
**zBMI at age 10**	-0.011 (0.018) *p* = 0.54	0.029 (0.019) *p* = 0.13	0.010 (0.018) *p* = 0.56

Model adjusted for age, sex, income sufficiency level, mother’s education at baseline, and whether participant was first born

Fit statstics: RMSEA = 0.052, CFI = 0.929, SRMR = 0.032

The longitudinal (with and without a 2-year lag) effects of parenting practices on BMIz in linear mixed models are presented (Table 3). Indeed from these mixed models, greater leniency and responsiveness increase the children’s BMI z-scores two years later. These results were consistent with the sensitivity analysis of parenting practices and the risk of having overweight/obesity (results not shown). In contrast, parenting style did not predict BMI z-scores in time-varying, linear mixed models. Parenting style with a two-year lag similarly did not predict BMI z-scores.

**Table 3:** Parenting practices and parenting styles on predicting children’s BMIz-scores in linear mixed models

	No lag^a^		2-year lag^a,b^	
Parameter	Estimate (SE)	*p*	Estimate (SE)	*p*
Model 1: Parenting practices				
Disciplinarian	0.01 (0.02)	0.38	-0.02 (0.02)	0.21
Lenient	0.01 (0.01)	0.59	0.03 (0.01)	0.04
Responsive	0.002 (0.02)	0.91	0.03 (0.02)	0.02
Model 2: Parenting styles				
Authoritative	Reference		Reference	
Authoritarian	0.02 (0.04)	0.60	-0.03 (0.03)	0.36
Uninvolved	-0.02 (0.03)	0.58	-0.04 (0.03)	0.17
Permissive	-0.003 (0.03)	0.92	0.02 (0.03)	0.49

^a^All models adjusted for age, sex, income sufficiency level, mother’s education at baseline, and whether participant was the first born

^b^Due to the nature of FIML preserving all observations, the first and last wave were omitted

## Discussion

To our knowledge, this is the first study to utilize a large, representative, longitudinal cohort with measured height and weight to assess the dynamic, bidirectional relationship between parenting practices and parenting styles with Canadian children’s BMI z-scores. Results support a dynamic relationship as the lagged effects from parenting practices differed across time as the child aged. This is consistent with one of the findings from a recent systematic review of prospective cohort studies investigating the relationships between parenting practices, parenting styles, and children’s weight [24]. For instance, in our study, lenient and responsive parenting practices were associated with lagged BMI z-score increases, but only when the child was younger. This may also explain why the psychometric properties of ‘permissive’ parenting practices when children were older were low. As children age, using standard measures of parenting practices based on younger children populations may not be reliable. In addition, perhaps rather than general parenting, measures that are focused on the context of food and feeding (such as parental feeding practices and parental feeding styles) are more pertinent to children’s obesity risk. Indeed, parental feeding style that is overly permissive (indulgent) is associated with increased children’s BMI z-scores and greater risk for obesity [25–28]. Importantly, while responsive feeding (such as recognizing a child’s satiety cues) is in theory part of the framework of broader responsive parenting practices and parenting styles [29], they are not always congruent with one another in practice [13, 30]. Relatedly, the literature has found that lenient households may have the most obesogenic home environments (such as greater availability of sugar-sweetened beverages and less restricted screen time) [31] but further research on this is needed.

The results from this study may additionally reflect how parenting practices and level of supervision changes based on the child’s developing autonomy and independence. Preliminary evidence from the literature suggests that the relationship between parent and child can be moderated by other factors, such as the child’s BMI. In contrast, we found no evidence of a bidirectional relationship between parenting practices and children’s BMI z-scores. Indeed, results suggested that the directionality of effects is mainly from parenting practices to zBMI, and the effect of children’s zBMI on parenting practices was minimal.

No significant relationships were detected when investigating parenting styles and BMI z-scores. While the cross-sectional literature has consistently reported relationships between parenting style and children’s BMI z-scores [32], the longitudinal studies are limited and mixed [9, 11, 33–35]. Many of these studies had follow-up of 4-years or less, had small samples, or samples that were not generalizable. In contrast, our study analyzed data from a large, representative sample with follow-up of six years. As our results suggest that parenting practices and parenting styles are dynamic and change as the child ages, the absence of ill health effects may be a reflection that indeed classifying parenting practices into four mutually exclusive parenting styles oversimplifies the relationship between parent and child. However, the bidirectionality and lagged effects of parenting styles and BMI z-scores or the risk of having overweight/obesity could not be fully investigated due to evidence of a singular covariance matrix for some of the parenting styles in the later waves. As this was a large, representative longitudinal cohort study with parenting style variability over time, only much larger studies may be able to address this methodological constraint.

This study is not without its limitations. With the exception of height and weight which were measured by study staff, many of the other relevant measures were based on parental report. Statistical approaches such as factor analysis and clustering were then used to identify parenting styles. While these are common approaches to identify latent constructs [36], the identification of the constructs are data-dependent. Nevertheless, these approaches were utilized as we were able to assess multiple constructs simultaneously. Alternative statistical approaches (such as latent class analysis) would have required us to analyze each construct separately and are also data-dependent. Direct observations of parenting style would have been more objective and definitive. However, such objective measures of parenting style over 16 years of follow-up would not have been feasible. As study participants completed questionnaires covering a broad range of topics lasting several hours every 1–2 years, concern for consistent, purposeful under- or over-reporting due to social desirability was diminished. Although consistent with other longitudinal studies, losses to follow-up were noted [37]. Thus we utilized FIML, a methodology that is mathematically equivalent to multiple imputation [38] to address missing data issues. The cohort is representative of Quebec youth, but may not be reflective of the entire Canadian youth population. In particular, Indigenous populations were not included in this cohort. Although Indigenous populations represent less than 5% of the Canadian population, they are especially vulnerable to obesity risk and should be further assessed in future studies. In particular, measures that are culturally sensitive and appropriate are needed as the existing parenting style measures may be overly focused on western societies and ideals.

We were unable to assess paternal parenting practices, nor the effects of convergent vs. divergent maternal and paternal parenting practices and parenting styles since only one parent (primarily mothers), completed the questionnaires. This also limited our ability to investigate the influence of, or comparison between parenting practices/styles to the family from a systems perspective. In particular, family functioning and home environment/organization may be related to the parent–child interactions and incorporate complementary/additional features of the home environment [39, 40]. Thus unmeasured confounding of the home environment as well as the larger social environment (including daycare and school) is likely and results should be interpreted with the scope of a single aspect of the social environment. Although we adjusted for socioeconomic status (via household income), unmeasured confounding through more proximal variables such as food insecurity or poor food availability may help explain the relationships and should be further studied. Although the study is a representative birth-cohort of Quebec children, race/ethnicity was relatively homogeneous. As the relationships may differ based on race/ethnicity and sex [35], future studies conducted among more diverse populations are needed. For instance, a recent systematic review noted the strength of association between food insecurity and children’s risk for having overweight or obesity was dependent on sex and age [41]. The cohort used for our analysis was originally created to investigate longitudinal predictors of academic performance. Thus, data pertinent to this secondary data analysis were more limited in scope. Future prospective cohort studies in which parenting practices, feeding styles, as well as the proximal variables of the home, school, and larger social environment are needed.

## Conclusions

By utilizing a large, prospective birth cohort of Quebec youth with 13 waves of data collection, this study is the first to use a representative sample of Canadian children to investigate the longitudinal associations between parenting practices, parenting styles and children’s zBMI and overweight/obesity risk. The complex, changing interactions between parent and child were incorporated by testing the bidirectional, time-varying, and lagged features into the analysis. Results indicate that the effects of parenting practices on children’s BMI z-scores are lagged and differential, but not bidirectional. As the home is the first and primary social environment for children, this study reinforces the impact of the parent–child dynamics that is critical to children’s health during the developmental years. Further research on preventive care targeted at the family system is needed.

## Data Availability

The data that support the findings of this study are available from the Québec Longitudinal Study of Child Development (QLSCD) research team. Restrictions apply to the availability of these data, which were used under license for the current study, and so are not publicly available. Further information on accessing data can be found on the website (https://www.iamillbe.stat.gouv.qc.ca/) or by contacting the QLSCD research team (iam_illbe@stat.gouv.qc.ca).

## References

[1] Rao DP, Kropac E, Do MT, Roberts KC, Jayaraman GC (2016). Childhood overweight and obesity trends in Canada. Health Promot Chronic Dis Prev Can Res Policy Pract.

[2] Sahoo K, Sahoo B, Choudhury AK, Sofi NY, Kumar R, Bhadoria AS (2015). Childhood obesity: causes and consequences. J Fam Med Prim Care.

[3] Reilly J, Methven E, McDowell Z (2003). Health consequences of obesity. Arch Dis Child.

[4] Juhola J, Magnussen CG, Viikari JS (2011). Tracking of serum lipid levels, blood pressure, and body mass index from childhood to adulthood: the Cardiovascular Risk in Young Finns Study. J Pediatr.

[5] Canning PM, Courage ML, Frizzell LM (2004). Prevalence of overweight and obesity in a provincial population of Canadian preschool children. CMAJ.

[6] Darling N, Steinberg L (1993). Parenting style as context: An integrative model. Psychol Bull.

[7] Baumrind D (1967). Child care practices anteceding three patterns of preschool behavior. Genet Psychol Monogr.

[8] Maccoby EE, Martin JA, Hetherington M (1983). Socialization in the context of the family: parent-child interaction. Handbook of Child Psychology.

[9] Olvera N, Power TG (2010). Brief report: parenting styles and obesity in Mexican American children: a longitudinal study. J Pediatr Psychol.

[10] Chen JL, Kennedy C (2004). Family functioning, parenting style, and Chinese children’s weight status. J Fam Nurs.

[11] Berge JM, Wall M, Loth K, Neumark-Sztainer D (2010). Parenting style as a predictor of adolescent weight and weight-related behaviors. J Adolesc Health.

[12] Gibson LY, Byrne SM, Davis EA, Blair E, Jacoby P, Zubrick SR (2007). The role of family and maternal factors in childhood obesity. Med J Aust.

[13] Hennessy E, Hughes SO, Goldberg JP, Hyatt RR, Economos CD (2010). Parent behavior and child weight status among a diverse group of underserved rural families. Appetite.

[14] Chen JL, Kennedy C (2005). Factors associated with obesity in Chinese-American children. Pediatr Nurs.

[15] Zeller MH, Boles RE, Reiter-Purtill J (2008). The additive and interactive effects of parenting style and temperament in obese youth seeking treatment. Int J Obes.

[16] Lande MB, Adams H, Falkner B (2009). Parental assessments of internalizing and externalizing behavior and executive function in children with primary hypertension. J Pediatr.

[17] Jetté M, Desgroseillers L. Survey description and methodology in the longitudinal study of child development in Quebec (QLSCD 1998–2002), Volume 1, No 1, Montréal: Institut de la Statistique du Québec; 2000.

[18] Shelton KK, Frick PJ, Wootton J (1996). Assessment of parenting practices in families of elementary school-age children. J Clin Child Psychol.

[19] Chao R, Willms JD. The effects of parenting practices on children’s outcomes. In: Vulnerable Children: Findings from Canada’s National Longitudinal Study of Children and Youth. Edmonton: University of Alberta Press; 2002.

[20] de Onis M, Onyango AW, Borghi E, Siyam A, Nishida C, Siekmann J (2007). Development of a WHO growth reference for school-aged children and adolescents. Bull World Health Organ.

[21] Minister of Industry. *L*ow Income Cut-Offs for 2007 and Low income measures for 2*006*. Available at: http://www.statcan.gc.ca/pub/75f0002m/75f0002m2008004-eng.pdf. Accessed October 24, 2022.

[22] Ursachi G, Horodnic IA, Zait A (2015). How reliable are measurement scales? External factors with indirect influence on reliability estimators. Procedia Econ Finance.

[23] Shrout PE, Fleiss JL (1979). Intraclass correlations: uses in assessing rater reliability. Psychol Bull.

[24] Sokol RL, Qin B, Poti JM (2017). Parenting styles and body mass index: a systematic review of prospective studies among children. Obes Rev.

[25] Hughes SO, Shewchuk RM, Baskin ML, Nicklas TA, Qu H (2008). Indulgent feeding style and children’s weight status in preschool. J Dev Behav Pediatr JDBP.

[26] Hughes SO, Power TG, O’Connor TM, Fisher JO, Micheli NE, Papaioannou MA (2021). Maternal feeding style and child weight status among Hispanic families with low-income levels: a longitudinal study of the direction of effects. Int J Behav Nutr Phys Act.

[27] Rodenburg G, Kremers SP, Oenema A, van de Mheen D. Associations of parental feeding styles with child snacking behaviour and weight in the context of general parenting. Public Health Nutr. Published online March 26, 2013:1–10. 10.1017/s1368980013000712 10.1017/S1368980013000712 PMC10282213 23527513

[28] Shloim N, Edelson LR, Martin N, Hetherington MM. Parenting styles, feeding styles, feeding practices, and weight status in 4–12 year-old children: A systematic review of the literature. Front Psychol. 2015;6. 10.3389/fpsyg.2015.01849 10.3389/fpsyg.2015.01849 PMC4677105 26696920

[29] Black MM, Aboud FE (2011). Responsive feeding is embedded in a theoretical framework of responsive parenting123. J Nutr.

[30] Melis Yavuz H, Selcuk B (2018). Predictors of obesity and overweight in preschoolers: The role of parenting styles and feeding practices. Appetite.

[31] Johnson R, Welk G, Saint-Maurice PF, Ihmels M (2012). Parenting styles and home obesogenic environments. Int J Environ Res Public Health.

[32] Berge JM (2009). A review of familial correlates of child and adolescent obesity: what has the 21st century taught us so far?. Int J Adolesc Med Health.

[33] Rhee KE, Lumeng JC, Appugliese DP, Kaciroti N, Bradley RH (2006). Parenting styles and overweight status in first grade. Pediatrics.

[34] Agras WS, Hammer LD, McNicholas F, Kraemer HC (2004). Risk factors for childhood overweight: a prospective study from birth to 9.5 years. J Pediatr..

[35] Fuemmeler BF, Yang C, Costanzo P (2012). Parenting styles and body mass index trajectories from adolescence to adulthood. Health Psychol Off J Div Health Psychol Am Psychol Assoc.

[36] Power TG (2013). Parenting Dimensions and Styles: A Brief History and Recommendations for Future Research. Child Obes..

[37] Galea S, Tracy M (2007). Participation rates in epidemiologic studies. Ann Epidemiol.

[38] Graham JW, Olchowski AE, Gilreath TD (2007). How many imputations are really needed? Some practical clarifications of multiple imputation theory. Prev Sci.

[39] Halliday JA, Palma CL, Mellor D, Green J, Renzaho AMN (2014). The relationship between family functioning and child and adolescent overweight and obesity: a systematic review. Int J Obes 2015.

[40] Carbert NS, Brussoni M, Geller J, Mâsse LC (2019). Familial environment and overweight/obese adolescents’ physical activity. Int J Environ Res Public Health.

[41] St Pierre C, VerPloeg M, Dietz WH (2022). Food insecurity and childhood obesity: A systematic review. Pediatrics..

